# Genome Sequence of *Novosphingobium lindaniclasticum* LE124^T^, Isolated from a Hexachlorocyclohexane Dumpsite

**DOI:** 10.1128/genomeA.00715-13

**Published:** 2013-09-12

**Authors:** Anjali Saxena, Namita Nayyar, Naseer Sangwan, Rashmi Kumari, J. P. Khurana, Rup Lal

**Affiliations:** Department of Zoology, University of Delhi, Delhi, Indiaa; Interdisciplinary Centre for Plant Genomics and Department of Plant Molecular Biology, University of Delhi South Campus, New Delhi, Indiab

## Abstract

*Novosphingobium lindaniclasticum* LE124^T^ is a hexachlorocyclohexane (HCH)-degrading bacterium isolated from a high-dosage-point HCH dumpsite (450 mg HCH/g soil) located in Lucknow, India (27°00′N and 81°09′E). Here, we present the annotated draft genome sequence of strain LE124^T^, which has an estimated size of 4.86 Mb and is comprised of 4,566 coding sequences.

## GENOME ANNOUNCEMENT

Sphingomonads represent a naturally selected taxon that can degrade and/or assimilate a wide range of xenobiotic compounds, including mono- and polycyclic aromatic compounds, chlorinated compounds, and pesticides (1). Members of this taxon are also known to be efficient degraders of hexachlorocyclohexane (HCH) isomers (1–3). In conformity with our primary objective, i.e., to elucidate the pangenomic variations across HCH-degrading genotypes, we have already sequenced two sphingomonads (4, 5). Both of their genomes represent species belonging to the genus *Sphingobium* (4, 5). We have sequenced the genome of yet another sphingomonad, belonging to the genus *Novosphingobium*, i.e., *N. lindaniclasticum* strain LE124^T^, isolated from an HCH dumpsite (6).

The genomic DNA of strain LE124^T^ was sequenced by the Illumina genome analyzer IIx platform (paired-end library, 2 kb, *n* = 4.96 × 10^8^, 90 bp/read) and the 454 GS FLX Titanium platform (single reads, *n* = 70,343,174, >350 bp). The draft genome sequence (4.86 Mb) of strain LE124^T^ was assembled (150× coverage) into 156 contigs (>500 bp ± 10 bp) using ABySS v.1.3.3 assembler (7), set at a *k*-mer size of 57. The final validated (based on paired-end criterion) assembly (N_50_ of contigs, 37.4 kb) (8) was annotated using RAST v.4.0 (9) and the NCBI Prokaryotic Genomes Automatic Annotation Pipeline v.2.1 (PGAAP) (http://www.ncbi.nlm.nih.gov/genomes/static/Pipeline.html), which predicted 4,566 coding sequences (average G+C percentage, 64.6).

RAST annotation (9) predicted 414 subsystems and 30 putative metal resistance proteins. Nine rRNAs, 54 tRNAs, and 28 pseudogenes were found using PGAAP. Additionally, 52 transposases, 40 ABC transporters, and 90 transcriptional regulators were found in the genome. BLASTp-based (10) comparison with the ISfinder database (11) reported 10 insertion sequence (IS) elements (IS*3/21*) assigned to *Sphingobium* spp. (4). Plasmid-related genes, i.e., *repA*, *par*, and conjugative transfer genes, were detected on contig 113 (77,158 bp). Average nucleotide identity (ANI) (12) analysis revealed that the draft genome of *N. lindaniclasticum* LE124^T^ is phylogenetically related to those of *Erythrobacter litoralis* (77.82%), *Novosphingobium aromaticivorans* (77.72%), and *Sphingopyxis alaskensis* (76.6%).

The HCH-degrading *lin* genes (13, 14) were found scattered throughout the draft genome assembly. A single copy of *linA* (dehydrochlorinase) was represented in the sequenced genome. Additionally, *linH*, *linK*, *linL*, *linM*, and two copies of *linG* were also present. In contrast to *Sphingobium indicum* B90A (4), *linB* (haloalkane dehalogenase) and *linDER* were absent from the genome of strain LE124^T^; this was also confirmed by PCR amplification. We have recently reported that HCH selection pressure is responsible for bringing *lin* genes through horizontal gene transfer (HGT) into sphingomonads (15), and these findings reflect that strain LE124^T^ has yet to acquire these genes through HGT.

Interestingly, the draft genome of strain LE124^T^ also showed the presence of a benzoate-degrading gene cluster with the presence of 4-hydroxybenzoate 3-monooxygenase, benzoate transporter proteins, and genes for the chloroaromatic catechol branch of the β-ketoadipate pathway. Additionally, genes encoding *ortho*-halobenzoate 1,2-dioxygenase alpha- and beta-intracellular serine protease (ISP) protein (*ohbA*, *ohbB*), known to be involved in xenobiotic and benzoate degradation, were also found. The genetic compendium required to resolve the intergenus-level genetic divergence of the *lin* genes and degradation pathway can be analyzed by doing comparative analyses of genomes of sphingomonads that have now been sequenced.

### Nucleotide sequence accession numbers.

This whole-genome shotgun project has been deposited at DDBJ/EMBL/GenBank under the accession no. ATHL00000000. The version described in this paper is version ATHL01000000.
